# The role of π-linkers and electron acceptors in tuning the nonlinear optical properties of BODIPY-based zwitterionic molecules†

**DOI:** 10.1039/d0ra02193h

**Published:** 2020-11-05

**Authors:** Tanushree Sutradhar, Anirban Misra

**Affiliations:** Department of Chemistry, University of North Bengal Darjeeling-734 013 West Bengal India anirbanmisra@yahoo.com anirbanmisra@nbu.ac.in +91-9434228745

## Abstract

Intramolecular charge transfer process can play a key role in developing strong nonlinear optical (NLO) response in a molecule for technological application. Herein, two series of boron dipyrromethene (BODIPY)-based push–pull systems have been designed with zwitterionic donor–acceptor groups, and their NLO properties have been evaluated using a density functional theory-based approach. Different π-conjugated linkers and electron acceptor groups were used to understand their roles in tuning the NLO properties. The molecules were analyzed through HOMO–LUMO gaps, frontier molecular orbitals, polarizabilities, hyperpolarizabilities, Δ*r* indices, transition dipole moment densities, ionization potentials, electron affinities and reorganization energies for holes and electrons. These observations correlated well with the computed absorption spectra of the molecules. It is found that with the introduction of different π-linkers in the molecule, planarity is maintained and the HOMO–LUMO gap is systematically decreased, which leads to a large NLO response. It was noted that the electronic absorption wavelength maxima were found in the near-infrared region (934–1650 nm). The results show that compared to the pyridinium acceptor group, the imidazolium acceptor group in the BODIPY systems amplifies the NLO response to a larger extent. It is also observed that the BODIPY-based dye with an imidazolium acceptor and thienothiophene π-linker shows the highest first hyperpolarizability value of 3194 × 10^−30^ esu. Furthermore, the charge transfer occurs in the *z*-direction, as the *z*-component of the first hyperpolarizability is the dominant factor in this system. Here, the designed molecules show a characteristic reorganisation energy value, which is a deciding factor in the rate of hole/electron transport for favourable intermolecular coupling. As a whole, this theoretical work highlights that π-conjugated linkers and electron acceptor groups can be used judiciously to design new molecular systems for optoelectronic applications.

## Abstract 2

BODIPY-based zwitterionic molecules with pyridinium and imidazolium electron acceptors and thienothiophene π-linkers reveal significant first hyperpolarizability.

## Graphical abstract

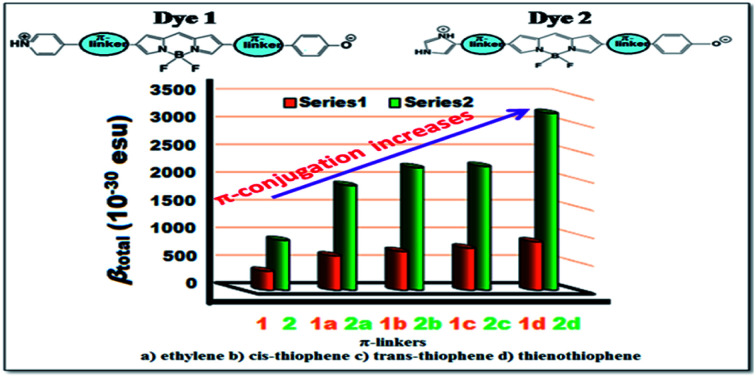

## 1. Introduction

Materials with high nonlinear optical (NLO) properties have gained much attention in recent years, as they offer potential applications in optoelectronic and photonic devices in the regulation of optical switching, micro fabrication and imaging, telecommunication, laser technology, data storage, *etc.*^1–9^ Different types of materials of both inorganic^10^ and organic^11–13^ origin have been investigated for their interesting nonlinear optical properties. However, among these, organic materials with high NLO response have proven to be the optimum choice due to their tunability.^7,14^ Most organic NLO materials are push–pull molecules containing π-conjugated systems linking an electron donor (D) to an electron acceptor (A) group.^15^ The NLO properties in these compounds are developed due to high polarization of π electrons along the conjugated backbone. The first hyperpolarizability (*β*) is associated with the intramolecular charge transfer (ICT) from the donor group to the electron acceptor group. Several studies confirmed that by optimizing the donor and acceptor groups, π-linker and ring twisting in the push–pull system, the ICT process can be amplified and hence the NLO response increases.^16–18^ The main strategies for designing efficient NLO materials are based on the choice of suitable donor and acceptor groups and π-conjugated bridges.^19,20^ Albert *et al.* suggested an approach in which by introducing zwitterionic behaviour in a conjugated molecule, one can enhance the NLO properties by providing low energy charge transfer.^21,22^ Some researchers also revealed that zwitterionic D and A groups positioned at opposite ends of the conjugated systems of chromophores can ameliorate their NLO response.^12,23^ Xiong *et al.* synthesized a thermally stable zwitterionic picolinium(dicyano) esterquinodimethane chromophore with a *β* value of 1800 × 10^−30^ esu.^24^ All these facts prompted us to design and investigate zwitterionic systems crafted by different donors and acceptors with high NLO response.

**Fig. 1:**
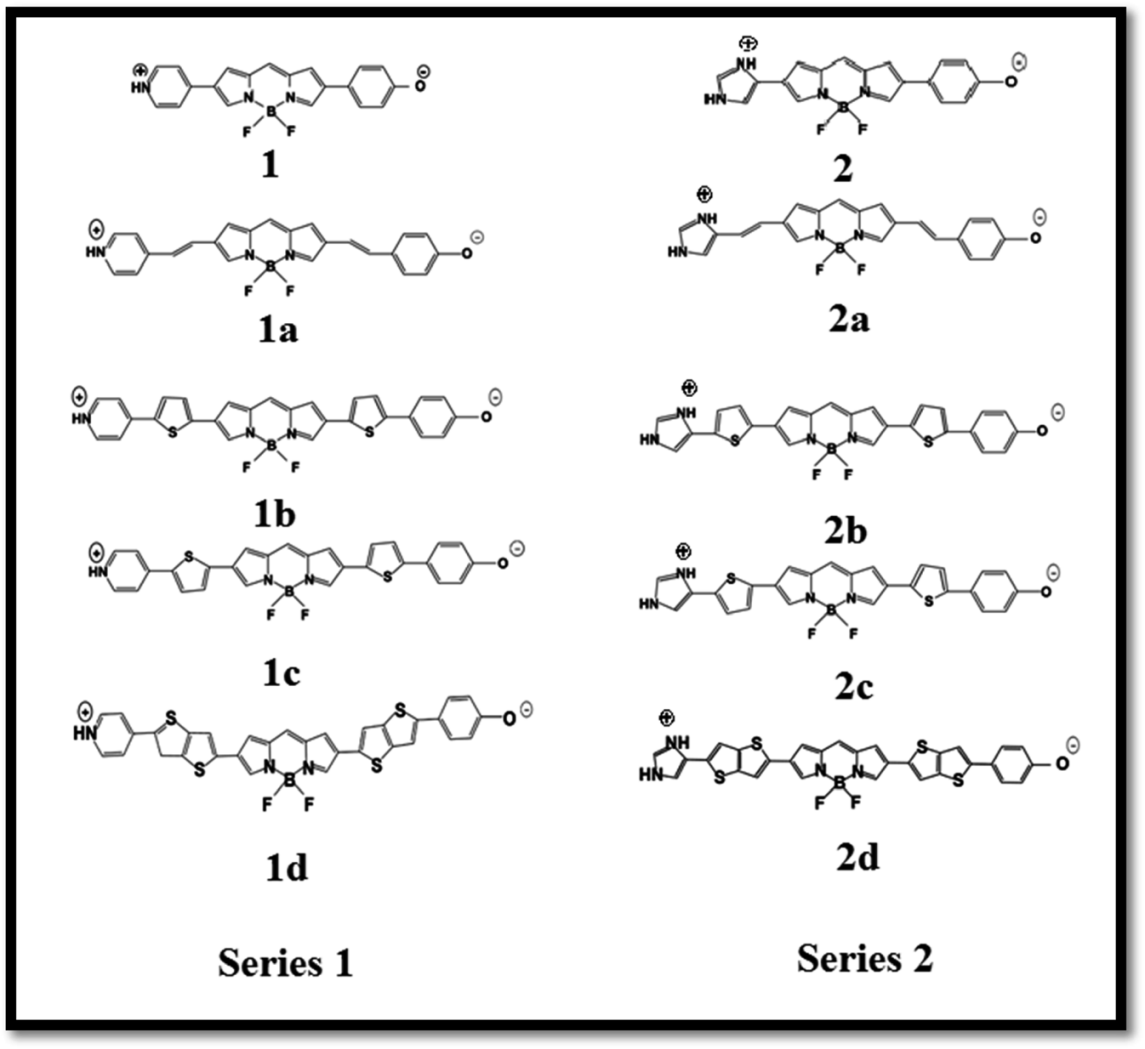
Chemical structures of the BODIPY-based dyes under investigation.

The boron dipyrromethene (BODIPY)-based push–pull chromophore is an area of recent interest and has been used in the present investigation. BODIPY dyes are well known for their desirable photophysical properties, as they possess strong absorption bands in the UV-vis region, high fluorescence quantum yields,^25,26^ two-photon absorption properties, *etc.*^27^ BODIPY dyes are widely used in photodynamic therapy,^28^ as chemosensors,^29^ fluorosensors,^30^*etc.* Extensive research has been performed on the molecular structures and optical properties of BODIPY dyes; however, very few studies have reported BODIPY-based push–pull zwitterionic systems. In this work, the main strategy is the introduction of donor and acceptor groups to the opposite ends of the BODIPY core at the 2 and 6 positions. Imidazolium compounds are known for their significant absorption in the entire UV region and for their fluorescence properties.^31,32^ It is reported that the imidazolium cation provides a remarkable contribution to enhanced NLO response.^33,34^ To investigate the effects of different electron acceptors and π-conjugated linkers on the NLO properties of the BODIPY system, 10 new BODIPY-based D–π–A dyes were designed (Fig. 1). Pyridinium and imidazolium ions were taken as positively charged acceptor groups, and phenoxide group was taken as the negatively charged donor group. Four π-spacers, ethylene, *cis*-thiophene with respect to BODIPY, *trans*-thiophene with respect to BODIPY and thienothiophene, were used as the 1^st^ and 3^rd^ π-linkers, and BODIPY was used as the 2^nd^ π-linker.

This paper deals primarily with the theoretical calculation of the optoelectronic properties of newly designed molecules using density functional theory (DFT)-based methods. The focus of our work was to determine the influence of different π-conjugated linkers and π-acceptors on the NLO properties of molecules. DFT and TDDFT calculations were carried out to evaluate the molecular properties, including the frontier molecular orbitals, absorption spectra, polarizability and hyperpolarizability, ionization potential (IP), electron affinity (EA) and reorganization energy of the hole and electron. It is exhibited that with increasing π-electron delocalization and substitution of pyridinium cation by imidazolium cation in the BODIPY-based systems, the NLO response is amplified. The present work will provide a direction to researchers for the synthesis of novel NLO materials of BODIPY-based dyes for technological application.

## 2. Theoretical background and computational details

Geometry optimization of the designed molecules was performed in the gas phase using the Gaussian 09 program at the B3LYP/6-311++G(d,p) level of theory.^35^ The molecules were optimized at the minimum energy level and no imaginary frequency was found, which signifies the stationary point of the minima. All the obtained restricted B3LYP solutions were stable. The B3LYP and CAM-B3LYP functionals were found to be the most suitable for NLO calculations. Recently, Prakasam *et al.* investigated the second order hyperpolarizabilities and absorption properties of triphenylamine-based organic sensitizers with the B3LYP/6-311++G(d,p) method.^36^ However, the B3LYP functional sometimes fails to predict accurate values in the case of polarizability of long chain molecules, charge transfer excitation, *etc.* Increasing the incorporated HF exchange fraction as well as the long range correction becomes significant to improve the results, and many researchers have attempted to achieve this. Very recently, Avramopoulos and co-workers designed a photochromic material with switchable nonlinear optical properties by employing the density functional method with the CAM-B3LYP functional.^37^ For this reason, we considered the B3LYP and CAM-B3LYP functionals for explanation of the NLO behaviour of the studied system. Hence, in order to analyze the NLO behaviour of the designed D–π–A systems, the dipole moment (*μ*_total_), average polarizability (*α*) and first hyperpolarizability (*β*) were evaluated using the B3LYP functional as well as the CAM-B3LYP^38^ functional with the 6-311++G(d,p) basis set to include a long range correction for improving the charge transfer characteristics in the Gaussian 09 software.^39^

The dipole moment was calculated using the following equation:1
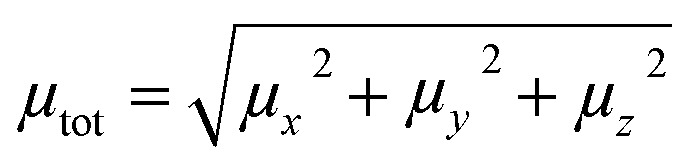
where *μ*_*x*_, *μ*_*y*_, and *μ*_*z*_ are the components of the dipole moments in the *x*, y, and *z* directions, respectively.

Average polarizability (〈*α*〉) was calculated using the following equation:^40^2
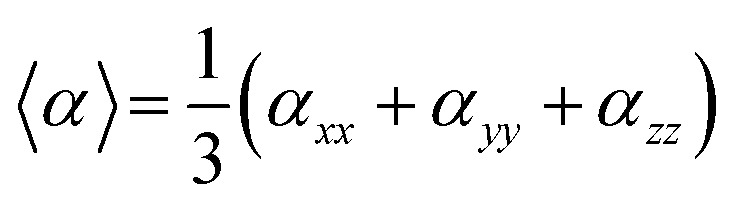
where *α*_*xx*_, *α*_*yy*_ and *α*_*zz*_ are the polarizability tensor components.

The first hyperpolarizability (*β*_total_) can be expressed as^40^

3 *β*_total_ = (*β*_*x*_^2^ + *β*_*y*_^2^ + *β*_*z*_^2^)^½^ = [(*β*_*xxx*_ + *β*_*xyy*_ + *β*_*zzx*_)^2^ + (*β*_*yyy*_ + *β*_*yzz*_ + *β*_*yxx*_)^2^ + (*β*_*zzz*_ + *β*_*zxx*_ + *β*_*zyy*_)^2^]^1/2^

where *β*_*xxx*_, *β*_*xyy*_, *β*_*zzx*_, *β*_*yyy*_, *β*_*yzz*_, *β*_*yxx*_, *β*_*zzz*_, *β*_*zxx*_ and *β*_*yyy*_ are hyperpolarizability tensors along the *x*, *y* and *z* directions, respectively.

The transition dipole moment (TDM) density has been evaluated on the basis of the dipole moment integral between the occupied and virtual molecular orbitals. The TDM density can be calculated and visualized using the Multiwfn wave function analyzer.^41^ The charge transfer length during electron excitation in the D–π–A system is defined as the Δ*r* index. This is defined by the following equation:^42^4
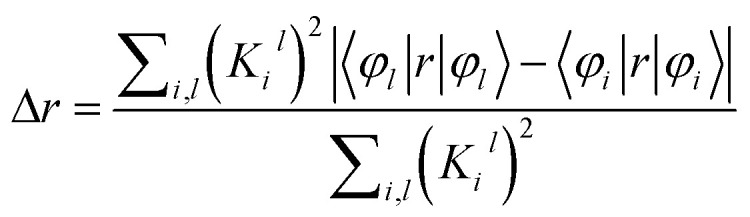
where the *i* and *l* indices run over all the occupied and vertical MOs, respectively, and *Φ* is the orbital wave function:

5 *K*_*i*_^*l*^ = *X*_*i*_^*l*^ + *Y*_*i*_^*l*^

where *X*_*i*_^*l*^ and *Y*_*i*_^*l*^ denote the configuration coefficients corresponding to *i*→*l* excitation and *l*→*i* de-excitation, respectively. The Δ*r* indices were calculated using Multiwfn software.

The ionization potential (IP) defines the energy changes due to removing electrons from or adding holes to the neutral molecule. On the other hand, the electron affinity (EA) quantifies the energy changes for adding electrons to or removing holes from the same. This can be expressed as:^43^

6 IP = *E*_Cation_ − *E*_Neutral_ and EA = *E*_Neutral_ − *E*_Anion_

*E* denotes the energy of the respective system. The hole transport reorganization energy (*λ*_hole_) and electron transport reorganization energy (*λ*_electron_) are calculated as:^44^

7 *λ*_hole_ = *λ*_1_ + *λ*_2_ = (*E*_0_^+^ − *E*_+_) + (*E*_+_^0^ − *E*_0_)

8 *λ*_electron_ = *λ*_3_ + *λ*_4_ =(*E*_0_^−^ − *E*_−_) + (*E*_−_^0^ − *E*_0_)

Here, *E*_0_, *E*_+_ and *E*_*−*_represent the energies of the neutral, cation and anion species, respectively, in their optimized geometries. *E*_0_^+/−^ and *E*_+/−_^0^ represent the energies of the cation/anion with the optimized structure of the neutral species and the energy of the neutral species with the optimized structure of the cation/anion geometry, respectively.

## 3. Results and discussion

### Electronic structure

The electronic structures of molecules play a key role in regulating the nonlinear optical properties of the molecules, as they depend on the geometry of the molecular system. Here, Fig. 1 presents the BODIPY molecules with different π-linkers and acceptors and a phenoxide donor. The optimized structures of the molecules, dihedral angles and bond angles are given in the ESI (Fig. S1–S10 and Tables S1–S10†). For a smooth electron transfer process from the donor unit to the acceptor unit, the dye must have co-planarity between the donor unit-bridging unit-acceptor unit (D–π–A).^45^ It is found that most of the dyes maintain co-planarity, as the dihedral angles are close to 0° and 180° and the bond angles are close to 120°. It was also noted that dye 2d has perfect planar geometry, as the geometrical parameters are very close to ideal. Thus, we can say that the inclusion of different π-linkers favours planarity in the investigated systems. It is reported that upon attaching an unsubstituted phenyl ring to the BODIPY core, the fluorescence quantum yield (*φ*_f_) decreases compared to that of BODIPY alone. Meanwhile, incorporation of two methyl groups in the BODIPY system restricts the motion of the phenyl ring and increases the *φ*_f_ of the molecule.^46^ Therefore, we can expect that incorporation of proper π-linkers will increase the NLO response.

The charge transfer process is highly dependent on the energy difference between the HOMO and LUMO (Δ*E*). Frontier Molecular Orbital (FMO) theory helps to predict the chemical stability of a molecule.^47,48^ Usually, the LUMO defines the capacity of acceptance of electrons and the HOMO classifies the electron donating ability.^49^ A low Δ*E* value indicates a chemically soft molecule, whereas a high Δ*E* value signifies a chemically hard molecule. Polarizability can be enhanced with increasing softness in a molecule, which facilitates the NLO response.

**Fig. 2:**
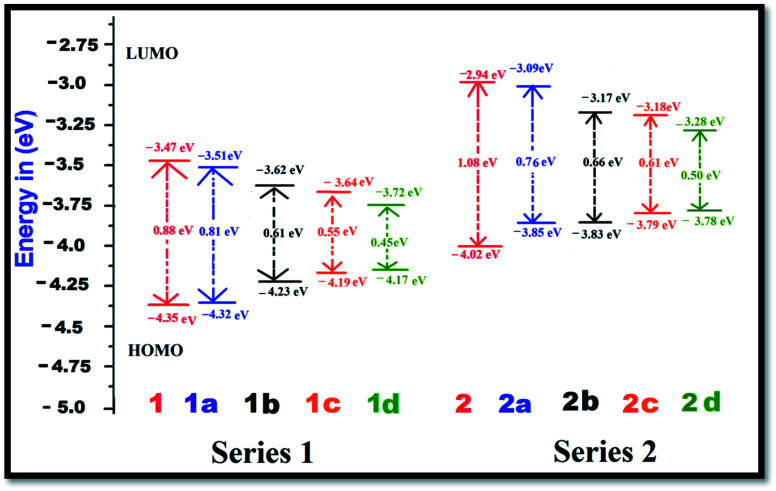
Energy level diagrams of the dyes in the gas phase at the B3LYP/6-311++G(d,p) level of theory.

**Fig. 3:**
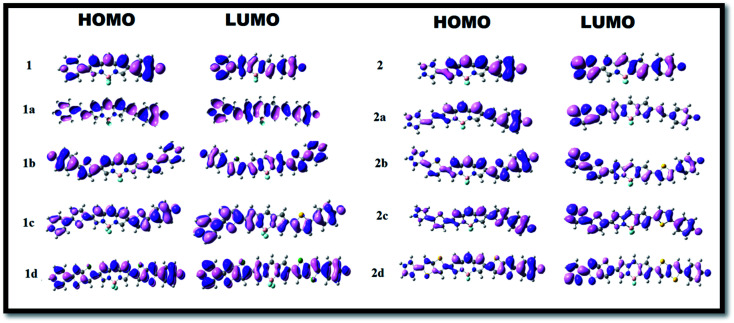
Frontier molecular orbitals of the dyes in the gas phase at the B3LYP/6-311++G(d,p) level of theory.

The FMO energy levels along with the HOMO–LUMO gaps are represented in Fig. 2. Proper inclusion of acceptor and donor units at the 2 and 6 positions of BODIPY endow the molecule with zwitterionic character, and the HOMO–LUMO energy gap is found to be reduced. Currently, the compounds which are studied can be categorized into two sets, namely, BODIPY-based pyridinium acceptors (Series 1) and imidazolium acceptors (Series 2). Upon introduction of different π-linkers in the BODIPY framework in series 1 and 2, Δ*E* starts to diminish from the respective values of 0.88 eV and 1.08 eV in a very systematic way. Upon insertion of –CH=CH– (1a and 2a), *cis*-thiophene (1b and 2b) and *trans*-thiophene (1c and 2c) groups in the BODIPY system, Δ*E* decreases successively. However, when thienothiophene groups are inserted (dye 1d and 2d), the lowest Δ*E* values of 0.45 eV and 0.50 eV are found. This clearly reveals that the thienothiophene π-linkers reduce the HOMO–LUMO gap more in the imidazolium acceptor system than in the pyridinium acceptor system. It is expected that by maintaining planarity, extended conjugation and a low Δ*E* gap, the molecules should achieve high NLO response. For some cases, the energy differences between the HOMO and LUMO of the designed dyes are very small. If we go along a series, the architecture of the molecule remains the same; only the conjugation length is increased, and as a consequence, the LUMO is stabilized. This could be the reason for these small differences. They also may be due to the limitations of the method used. Zhang *et al.* synthesized and performed DFT-based B3LYP calculations on a series of highly polarizable bis(*N*,*N*-diethyl) aniline-based chromophores. In this study, the reported energy differences of the compounds are also very low.^50^ Therefore, these values can be taken as an explanation of the NLO behaviour of the investigated compounds. The orders of the HOMO–LUMO energy gaps of series 1 and 2 are 1d < 1c < 1b < 1a < 1 and 2d < 2c < 2b < 2a < 2. Therefore, it can be predicted that all these molecules will show higher absorption wavelengths and will be excellent candidates for NLO materials for various photoelectronic applications. The modification of the π-conjugated linker by tailoring the structure would be a strategy to obtain high NLO activity. In order to understand the distribution patterns of the HOMOs and LUMOs, we analyzed the frontier molecular orbitals of the investigated dyes, which are given in Fig. 3. From the FMO diagram, it is evident that for Series 1, the HOMOs and LUMOs of all the chromophores are delocalized throughout the molecules. Meanwhile, for Series 2, the HOMO is localized on the phenoxide donor part and π-conjugated part of the molecule, and the LUMO is localized mainly on the imidazolium acceptor part. This fact signifies the electron donating ability of phenoxide ion and the electron acceptance nature of imidazolium cation. Overall, the calculated geometrical parameters indicate that planarity and delocalization in a molecule can be adjusted by varying the π-linkers, which are significant characteristics for tuning optoelectronic properties.

### NLO properties

To evaluate the NLO properties of the dyes, it was necessary to assess the magnitudes of the static dipole moment, linear response (polarizability) and nonlinear response (1^st^ hyperpolarizability). Benchmarking of the computational model for the NLO calculation was performed with some well-known push–pull systems given in the ESI^51^ (Table S14†). A comparison between the theoretically calculated results and experimental results of the hyperpolarizability show good agreement. The benchmarking exercise demonstrates that the hyperpolarizability value for the B3LYP functional is closer to the experimental value compared to that for the CAM-B3LYP functional. Here, our main objective was to find the effects of the zwitterionic donor–acceptor group and π-linkers on the hyperpolarizability value. Therefore, to obtain an explanation for the changes in the hyperpolarizability values with the structural modification in the framework of the donor–acceptor molecule in the gas phase, we kept the other tuning parameters unchanged. Hence, to determine the influence of the π-conjugated linkers and electron acceptor groups on the NLO properties of the Series 1 and Series 2 compounds, these parameters were calculated with the B3LYP and range-separated CAM-B3LYP functionals with a standard basis set, 6-311++G(d,p), and the results are tabulated in Tables 1 and S12 (ESI†). In the following discussion, we illustrate the results obtained using the B3LYP functional. The average polarizability (Δ*α*) of the dyes increases in the order of *a* < *b* < *c* < *d* for both series, and the results found using the B3LYP and CAM-B3LYP functionals follow the same trend. Looking at Table 1, one can find that the average polarizabilities of dye 1 and 2 are 103.37 × 10^−24^ esu and 83.72 × 10^−24^ esu, respectively. With the addition of ethylene π-linkers as the 1^st^ and 3^rd^ conjugators in the 1 and 2 dyes, Δ*α* increases by 61 × 10^−24^ esu and 65 × 10^−24^ esu, respectively. The polarizability further increases with the addition of the thiophene π-linker at the *cis* and *trans* positions of BODIPY. It was found that the average polarizabilities reached maxima and the values increased by 242 × 10^−24^ esu and 238 × 10^−24^ esu, respectively, when thienothiophene groups were incorporated in the parent compounds (Table 1). Therefore, Tables 1 and S12† indicate that with modification by different π-conjugated linkers, the polarizability is enhanced systematically. The highest polarizability was found for dye 1d, 345.52 × 10^−24^ esu, with the smallest Δ*E* (Table 1). It is known that a small energy gap between the HOMO and LUMO influences the polarizability of a molecule, and the results are in accordance with the calculated HOMO–LUMO gaps.

**Table 1:** Dipole moments, static polarizabilities and first hyperpolarizabilities of the studied dyes at the B3LYP/6-311++G(d,p) level of theory

Molecules	*μ*, debye	Δ*α*, 10^−24^ esu	*μβ*, 10^−48^ esu
1	27.24	103.37	9359
1a	31.50	163.64	19 561
1b	34.19	230.47	23 968
1c	35.34	234.66	26 900
1d	41.22	345.52	36 058
2	33.63	83.72	30 308
2a	42.40	147.55	80 177
2b	46.53	208.24	102970
2c	48.16	211.91	107734
2d	57.28	320.46	182951

**Fig. 4:**
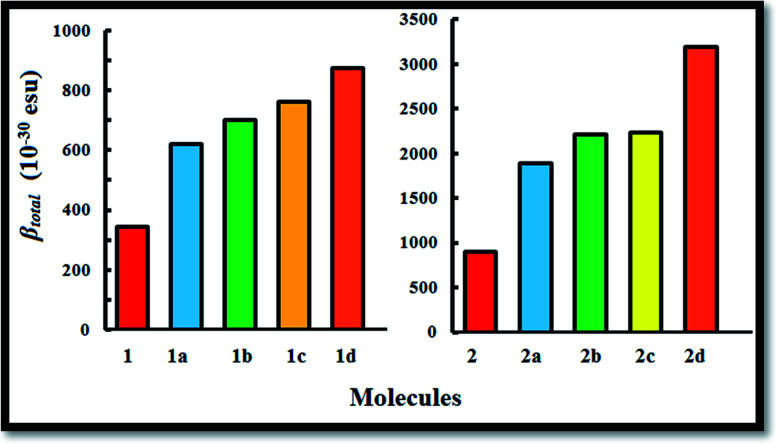
First hyperpolarizabilities of BODIPY-based dyes studied at the B3LYP/6-311++G(d,p) level of theory.

The first hyperpolarizability is connected with the ICT process. This is due to the flow of electron density from D to A *via* a π-conjugated bridge, which is discussed in more detail later. Among the investigated molecules, the dipole moment was found to be highest for dye 2d, while the lowest value was found for dye 1. It is reported that a large dipole moment and hyperpolarizability play significant roles in poled polymers.^52^ Therefore, the high dipole moment of these molecules is likely to enhance the NLO response. The first hyperpolarizability is graphically represented in Fig. 4. Calculation of the *β*_total_ value using the B3LYP and CAM-B3LYP functionals follows the same trend of increasing order. An increase in the *β*_total_ value of 557 × 10^−30^ esu is observed in the case of the imidazolium acceptor group (dye 2) compared to the pyridinium acceptor (dye 1), which proves the better electron acceptance power of imidazolium cation.^34^ This is also found in the frontier molecular orbitals. Changes in the π-conjugation length due to the inclusion of the 1^st^ and 3^rd^ π-linkers affect the NLO properties of the studied systems. For Series 1 and Series 2, with the incorporation of the ethylene π-linker, the *β*_total_ values are almost doubled. It was found that with the addition of thio-linkers in both series, the 1^st^ hyperpolarizability increased systematically. Compared to the *cis*-thiophene π-linkers, the *trans*-thiophene π-linkers improved the *β*_total_ value by 60 × 10^−30^ esu in dye 1c and 24 × 10^−30^ esu in dye 2c. When thienothiophene molecules were added to dyes 1 and 2, the *β*_total_ value increased by 531 × 10^−30^ esu and 2293 × 10^−30^ esu, respectively. This can be correlated with the HOMO–LUMO gaps of the molecules, as shown in Fig. 2. It is known that the 1^st^ order hyperpolarizability increases with decreasing Δ*E*.^53,54^ If we compare Fig. 2, 4 and Table 1, it can be found that for all cases, the Δ*E* gap is inversely proportional to the dipole moment, linear polarizability and 1^st^ order hyperpolarizability. The *β*_total_ values of the two series decrease in the orders of 1d > 1c > 1b > 1a > 1 and 2d > 2c > 2b > 2a > 2 for the B3LYP as well as the CAM-B3LYP functional. It was observed that BODIPY-based dye 2d, which contains an imidazolium acceptor and thienothiophene π-linker, shows the highest *β*_total_ and *μβ* value of 3194 × 10^−30^ esu and 18.29 × 10^−44^ esu, respectively. This NLO response can be attributed to the effective charge transfer from the D to the A moiety. The computed *β*_total_ value of 2d is 4110 times higher than the 1^st^ hyperpolarizability value of urea, which is used as a reference in organic systems. It should also be noted that in both series, the order of the *β*_total_ value is in conformity with the average polarizability. For better insight, we report the components of the 1^st^ hyperpolarizability in Tables 2 and S13† (ESI†). Yu *et al.* argued that *β*_*y*_ controls the *β*_total_ of 2,3-naphto-15-crown-5 ether for some metal cation complexes.^55^ They conclude that during the polarization process, the charge flows in the *y* direction. The results in Tables 2 and S13† indicate that for the investigated systems, *β*_total_ is dominated by the *β*_*z*_ component. The contribution of *β*_*x*_ is negligible. Therefore, we can surmise that during the polarization process, the maximum charge is expected to transfer along the *z* direction.^56^

**Table 2:** *β*_*x*_, *β*_*y*_, and *β*_*z*_ components (10^−30^ esu) of the studied molecules (1 to 2d) obtained from DFT calculations (B3LYP functional) employing the 6-311++G(d,p) basis set

Molecules	*β*_*x*_	*β*_*y*_	*β*_*z*_
1	−9.84	83.69	333.09
1a	11.0	146.99	603.38
1b	−4.45	83.37	696.03
1c	−0.296	72.43	757.71
1d	−12.7	155.17	860.81
2	−6.39	102.47	895.35
2a	15.4	−243.53	1875.16
2b	6.38	70.57	2212.42
2c	48.2	−309.23	2215.48
2d	−16.4	−417.39	3166.54

**Fig. 5:**
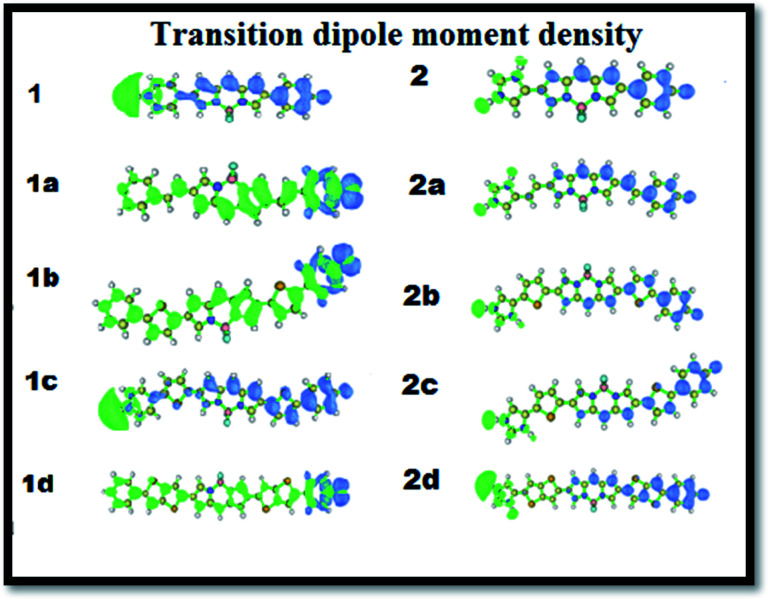
The transition dipole moment density of the studied molecules (1 to 2d). The isovalue is 2 × 10^−4^. The green colour implies charge increase upon excitation and the blue colour implies charge depletion.

To explain the origin and variation of the major transition energy of the NLO response, the Δ*r* index, integral overlap of hole electron distribution (*s*) and distance between the centroid of the hole and electron (*D*) were tabulated (Table 3). From Table 3, we can find that for all the cases, the Δ*r* index is larger than 2.0; hence, they may be regarded as charge transfer (CT) mode.^42^ If we compare Fig. 4 and Table 3, we can find that both the *β*_total_ and Δ*r* indices increase, with the π-conjugated linkers showing the same trend of increment. The 2d compound shows the highest Δ*r* indices and was found to show the highest *β*_total_ values. If we compare these two series, it is exhibited that for both series, the thienothiophene π-conjugated linkers show the highest Δ*r* indices, and the imidazolium acceptor system proves to be better for charge transfer. This can be well correlated with the 1^st^ hyperpolarizability of the molecules. The integral overlap of the hole electron distribution (*s*) is a tool to measure the spatial separation of holes and electrons and the distance between the centroids of holes and electrons (*D*), which define the charge transfer length. The higher the value of *D*, the greater the charge transfer length. For CT transition mode, *D* should be large and *s* should be small. The smallest *s* value of 0.015 and highest *D* value of 21.04 Å were found for dye 2d, which elucidates the genesis of the highest NLO response. The transition dipole moment densities are plotted in Fig. 5.

**Table 3:** Major transition energies, Δ*r* indices, integral overlaps of hole electron distribution (*s*) and distances between the centroid of the hole and electron (*D*) calculated at the CAM-B3LYP/6-311++G(d,p) level of theory

Compound	State	Transition energy (eV)	Δ*r* (Å)	*s*	*D* (Å)
1	S5	2.915	12.332	0.042	11.33
1a	S3	2.714	12.261	0.162	7.51
1b	S5	2.566	14.107	0.146	9.63
1c	S5	2.742	17.394	0.136	16.29
1d	S5	2.436	17.801	0.135	13.47
2	S3	2.449	12.117	0.024	10.73
2a	S4	2.129	15.228	0.018	13.89
2b	S4	1.953	17.563	0.017	16.42
2c	S4	1.889	17.846	0.017	16.76
2d	S4	1.734	21.805	0.015	21.04

### UV-vis spectra of dyes

Time-dependent density functional theory (TDDFT) was used to calculate the vertical excitation energies of the designed dyes. TDDFT computations were carried out using the B3LYP and CAMB3LYP functionals with the 6-311++G(d,p) basis set. The TDDFT method is a very popular choice for the calculation of vertical excitation energy. However, it has some limitations when predicting the transition energies for charge transfer-based molecules.^57^ Jacquemin and co-workers performed computational analysis of the spectral properties of BODIPY-derived systems with different functionals using the TDDFT methodology^58^ and compared the results with experimental data. In this paper, they also designed new aza-BODIPY molecules for near-infra red (NIR) applications with the same methodology. For the theoretical calculation of the vertical excitation energy of BODIPY-based molecules, TDDFT was found to be a suitable choice. Therefore, here, we used TDDFT to calculate the absorption spectra of the designed dyes absorbing light in the NIR region. A long-range correlated functional such as CAM-B3LYP is a better choice to calculate the vertical excitation energy to understand the charge transfer characteristics in molecules.^59,60^ Benchmarking of the TDDFT methodology was performed with some common BODIPY systems, which are represented in Table 4.^25^ The correlation of computed and experimental results induced us to choose the CAM-B3LYP functional for the estimation of the vertical excitation energy of our designed systems. The vertical excitation energies, maximum absorption wavelengths, oscillator strengths and nature of the transitions are reported in Tables 5 and S11 (ESI†). When we compare Fig. 2, Tables 5 and S11,† we can find that the excitation energies in the case of the CAM-B3LYP functional are in consonance with the HOMO–LUMO gaps of the molecules. For most of the cases, the differences found in the excitation energy calculations with the B3LYP and CAM-B3LYP functionals varied by about ∼0.2 eV; this is due to the different functionals of DFT. In the following discussions, the results are illustrated by taking the CAM-B3LYP functional. In all cases, the S_0_ → S_1_ electronic transitions dominated with high oscillator strength, and for dyes 1b, 1c and 1d, the S_0_ → S_2_ electronic transitions were found to have moderate oscillator strength. The excitation energies of dye 1 and dye 2 are 1.173 eV and 1.327 eV, respectively. From these results, it was found that upon substitution of the pyridinium acceptor by the imidazolium acceptor in the BODIPY system, the absorption wavelengths blue shifted due to the higher HOMO–LUMO gap compared to that of the pyridinium acceptor. Table 5 indicates that when an ethylene group is included in the D–π–A system in dyes 1 and 2, the absorption maxima are red shifted by 29 nm and 88 nm, respectively. This is due to the increases of the π-conjugation length of the system. The excitation energy systematically decreases with addition of thiophene π-linkers. Compared to *cis*-thiophene, the *trans*-thiophene systems (1c and 2c) are red shifted by 128 nm and 143 nm. The maximum absorption wavelengths were found for dyes 1d (1650 nm) and 2d (1471 nm) when thienothiophene groups were added as the 1^st^ and 3^rd^ π-linker in the BODIPY donor–acceptor system, respectively. The structural modification in the BODIPY system pushes the chromophore to absorb light in the NIR region (933 nm–1471 nm). In both series, the absorption wavelengths red shifted with the addition of π-linkers to the parent compounds. The increasing orders of *λ*_max_ are 1 < 1a < 1b < 1c < 1d and 2 < 2a < 2b < 2c < 2d. The highest oscillator strength was found for dye 1d, which is 1.4957. Overall, it can be concluded that with the addition of different π-linkers to the D–π–A system, the *λ*_max_, oscillator strength, and ICT character can be systematically modified.

**Table 4:** TDDFT benchmarking of the theoretical and experimental maximum absorption wavelengths (*λ*_max_); *f* represents the oscillator strength

Molecule	Solvent	Experiment *λ*_max_ (nm)	Theoretical *λ*_max_ (nm)	
			B3LYP CAM-B3LYP/6-311++G(d,p)	
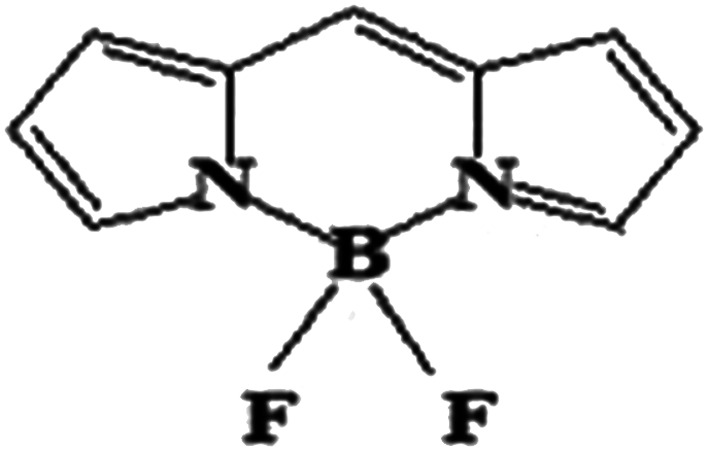	Methanol	497	415.4	418.1
			*f* = 0.5047	*f* = 0.5979
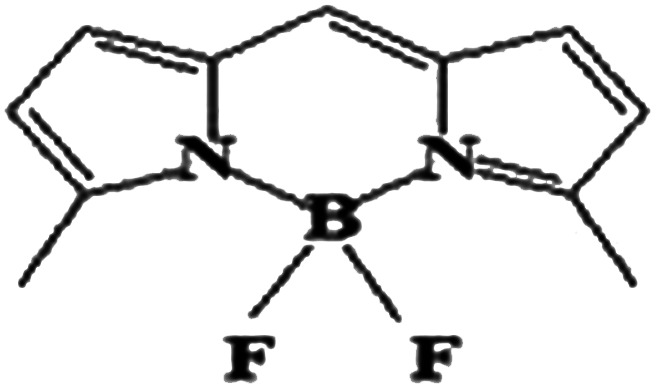	Ethanol	507	431.3	435.6
			*f* = 0.5986	*f* = 0.6579
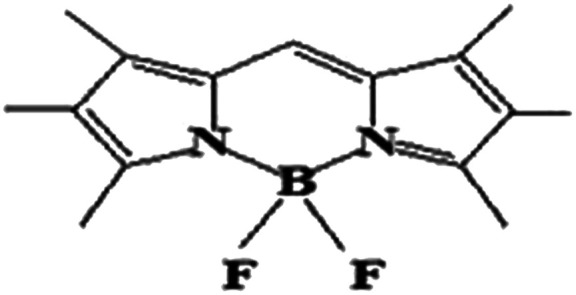	Ethanol	528	446.15	445.65
			*f* = 0.5978	*f* = 0.7163

**Table 5:** Main electronic transitions, maximum absorption wavelengths (*λ*_max_), oscillator strengths (*f*) and transition nature of BODIPY-based dyes in the gas phase at the CAM-B3LYP/6-311++G(d,p) level of theory

Dye	Excited energy (eV)	*λ*_max_ (nm)	*f*	Assignment	
1	1.173	1056.93	0.7333	H → L	0.667
1a	1.142	1085.95	1.3417	H → L	0.706
1b	0.932	1329.79	1.3891	H → L	0.737
	1.281	968.15	0.2467	H → L +1	0.689
1c	0.819	1512.20	1.4008	H → L	0.712
	1.218	1017.71	0.6243	H → L +1	0.657
1d	0.751	1649.98	1.4957	H → L	0.759
	1.128	1099.48	0.5036	H → L +1	0.683
2	1.327	933.96	0.4988	H → L	0.651
2a	1.214	1021.53	0.9052	H → L	0.539
2b	1.075	1153.25	1.1855	H → L	0.680
2c	0.957	1296.38	1.0327	H → L	0.655
2d	0.843	1471.35	1.052	H → L	0.775

### Ionization potential, electron affinity and reorganization energy

Isolated molecules with low reorganization energy are associated with high solid state charge carrier mobility.^61,62^ It was also found that some N-containing conjugated organic compounds are promising n-type or p-type materials for the fabrication of optical light emitting diodes (OLEDs).^63,64^ This arises due to their excellent optoelectronic properties, thermal stabilities, high electron mobilities, *etc.* As hyperpolarizability is associated with the charge transfer from the donor group to the acceptor group, determination of the ionization potential (IP), electron affinity (EA) and reorganization energy of organic molecules confers relevant information about the charge transport properties and charge injection character of a molecule. The adiabatic IP, EA and reorganization energy values of the ten molecules were calculated at the B3LYP/6-311++G(d,p) level and are represented in Table 6. For all the molecules, the potential energy of the cationic state is higher than the potential energy of the neutral state; this provides a positive ionization potential. In the case of an anion, the potential energy is lower than the potential energy of the neutral state, which gives a negative EA. The IP and EA values of the studied dyes are in the ranges of 5.37 eV to 4.47 eV and 2.04 eV to 3.05 eV, respectively. It is known that molecules with higher EA values show higher electron transport abilities and molecules with lower IP values show higher hole transport ability.^65^ Compound 2d has the lowest IP value of 4.47 eV, and 1d has the highest EA value of 3.05 eV. Therefore, molecules 1d and 2d will transport electrons and holes better than the other molecules. It is interesting to note that with the decrease of the HOMO–LUMO gap in both systems, the IP values gradually decrease and the EA values increase.

**Table 6:** Calculated ionization potentials (IPs), electron affinities (EAs), and reorganization energies (*λ*_hole_ and *λ*_electron_) of the dyes in the gas phase at the B3LYP/6-311++G(d,p) level of theory (in eV)

Molecule	IP	EA	*λ*_hole_	*λ*_electron_
1	5.37	2.45	0.39	1.35
1a	5.20	2.67	0.12	0.19
1b	5.01	2.88	0.09	0.20
1c	4.96	2.90	0.09	0.19
1d	4.85	3.05	0.08	0.17
2	5.13	2.04	0.14	1.12
2a	4.83	2.11	0.13	0.27
2b	4.65	2.34	0.17	0.18
2c	4.59	2.37	0.11	0.23
2d	4.47	2.81	0.06	0.21

To examine the effects of various substitutions on the reorganization energy for hole/electron transport (*λ*_hole/electron_), *λ*_hole_ and *λ*_electron_ are tabulated in Table 6. To obtain high efficiency optical light-emitting diodes, it is exigent to optimize the charge carrier (electron/hole) injection process and the electron–hole recombination process.^66^ Also, the charge injection and charge transport are crucial to ascertain the luminescence efficiency of OLEDs, and these can be obtained using a separate electron transport material and hole transport material.^67^ According to Marcus theory, the reorganization energy is the deciding factor in the rate of charge transfer, and this energy is required for structural changes associated with the charge transfer process.^68^ It is well known that the smaller the hole transport reorganization energy (*λ*_hole_), the better the hole transport properties, and the smaller the electron transport reorganization energy (*λ*_electron_), the better the electron transport properties. As expected, with increasing π-conjugation length, the *λ*_hole_ and *λ*_electron_ values decreased for most of the cases. This implies that the hole/electron transport properties increase along the series. The smallest *λ*_hole_ and *λ*_electron_ values were found for the thienothiophene-substituted linker in both series, which is in accordance with the NLO properties of the molecules. This can be explained by the increased rigidity and planarity of the molecules. The smallest *λ*_hole_ and *λ*_electron_ values were found for dye 2d and dye 1d, which can be correlated with the obtained IP and EA values of the studied molecules. Most interestingly, it was found that for all the cases, *λ*_hole_ is smaller than *λ*_electron_, which indicates that the molecules are better hole transporters than electron transporters. The differences between *λ*_hole_ and *λ*_electron_ of the compounds with π-spacers are less than 0.15 eV, which suggests that they can act as emitters with moderately high light–emitting efficiencies.^69^ Thus, the studied molecules are promising candidates as hole transport materials.

## 4. Conclusions

In the present computational study, two series of novel zwitterionic BODIPY-based molecules with pyridinium and imidazolium electron acceptors, a phenoxide unit as the electron donor and various π-linkers were designed, and their nonlinear optical properties were investigated. DFT-based methods were employed to explore the electronic structures, dipole moments, polarizabilities, hyperpolarizabilities, absorption properties, IPs, EAs and reorganization energies of these molecules. Our results reveal that with the introduction of different π-linkers in the D–π–A systems, planarity was maintained and the HOMO–LUMO gap systematically decreased. It was observed that thienothiophene groups reduced Δ*E* more than the other π-linkers. This observation was also supported by the excitation energy calculated with the TDDFT approach. Small HOMO–LUMO gaps of the molecules prompted the systems to absorb light in the NIR region. The FMO analysis depicts that for the Series 1 dyes, the electrons are delocalized throughout the molecules, whereas for the Series 2 dyes, the donor unit largely stabilizes the HOMO along with the π-linker and the LUMO is localized on the acceptor unit. The intramolecular charge transfer from the phenoxide unit to the imidazolium unit through the π-conjugated backbone plays a significant role in obtaining large NLO responses in the Series 2 dyes. The computed *β*_total_ values of the dyes were found to be 442 (for dye 1) to 4110 (for dye 2d) times greater than the value of urea molecule. Among the studied molecules, 2d exhibited the highest *β*_total_ value. The imidazolium acceptor showed better NLO response than the pyridinium acceptor. It was also noted that the dipole moment, polarizability and hyperpolarizability are consistent with the HOMO–LUMO gaps of the molecules. The lowest electron transport reorganization energy (0.17 eV) and highest EA were found for dye 1d, and the smallest hole transport reorganization energy (0.06 eV) and lowest IP were found for dye 2d. Consequently, 1d and 2d appear to be highly efficient emitters with promising hole transport characteristics. Overall, all the investigated dyes show high NLO response. The first hyperpolarizability of the molecules was found to respond dominantly in the *z* direction, which indicates the course of charge transfer. A good correlation was found between the Δ*r* index, TDM density, hyperpolarizability value and hole/electron transport properties. As a whole, this work demonstrates that the structural modification of π-linkers and electron acceptors in designing D–π–A systems is a significant approach to obtain high-performance NLO materials.

## Conflicts of interest

There are no conflicts to declare.

## Supplementary Material

RA-010-D0RA02193H-s001

## References

[cit1] Coe B. J. (2013). Coord. Chem. Rev..

[cit2] Marks T. J., Ratner M. A. (1995). Angew. Chem., Int. Ed. Engl..

[cit3] Lacroix P. G. (2001). Eur. J. Inorg. Chem..

[cit4] Peng Z., Yu L. (1994). Macromolecules.

[cit5] Tsutsumi N., Morishima M., Sakai W. (1998). Macromolecules.

[cit6] Breitung E. M., Shu C.-F., McMahon R. J. (2000). J. Am. Chem. Soc..

[cit7] Dalton L. R., Sullivan P. A., Bale D. H. (2009). Chem. Rev..

[cit8] Di Bella S. (2001). Chem. Soc. Rev..

[cit9] Majumder M., Goswami T., Misra A. (2018). ChemistrySelect.

[cit10] Roundhill D. M. and Fackler Jr J. P., Optoelectronic properties of Inorg. Compd., Springer Science & Business Media, 2013

[cit11] Sutradhar T., Misra A. (2019). ChemistrySelect.

[cit12] Majumder M., Misra A. (2018). Phys. Chem. Chem. Phys..

[cit13] Kölmel D. K., Hörner A., Castañeda J. A., Ferencz J. A., Bihlmeier A., Nieger M., Bräse S., Padilha L. A. (2016). J. Phys. Chem. C.

[cit14] Zyss J. , Molecular nonlinear optics: materials, physics, and devices, Academic press, 2013

[cit15] Sharipova S. M., Kalinin A. A. (2017). Chem. Heterocycl. Compd..

[cit16] Teran N. B., He G. S., Baev A., Shi Y., Swihart M. T., Prasad P. N., Marks T. J., Reynolds J. R. (2016). J. Am. Chem. Soc..

[cit17] Yang M., Jacquemin D., Champagne B. (2002). Phys. Chem. Chem. Phys..

[cit18] Khan M. U., Khalid M., Ibrahim M., Braga A. A. C., Safdar M., Al-Saadi A. A., Janjua M. R. S. A. (2018). J. Phys. Chem. C.

[cit19] Driessen A. , Nonlinear Optics for the Information Society, Springer, 2007

[cit20] Janjua M. R. S. A., Khan M. U., Bashir B., Iqbal M. A., Song Y., Naqvi S. A. R., Khan Z. A. (2012). Comput. Theor. Chem..

[cit21] Albert I. D., Marks T. J., Ratner M. A. (1997). J. Am. Chem. Soc..

[cit22] Albert I. D., Marks T. J., Ratner M. A. (1998). J. Am. Chem. Soc..

[cit23] Geskin V. M., Lambert C., Brédas J.-L. (2003). J. Am. Chem. Soc..

[cit24] Xiong Y., Tang H., Zhang J., Wang Z. Y., Campo J., Wenseleers W., Goovaerts E. (2008). Chem. Mater..

[cit25] Loudet A., Burgess K. (2007). Chem. Rev..

[cit26] Sutradhar T., Misra A. (2018). J. Phys. Chem. A.

[cit27] Zhang X., Xiao Y., Qi J., Qu J., Kim B., Yue X., Belfield K. D. (2013). J. Org. Chem..

[cit28] Kamkaew A., Lim S. H., Lee H. B., Kiew L. V., Chung L. Y., Burgess K. (2013). Chem. Soc. Rev..

[cit29] Gabe Y., Urano Y., Kikuchi K., Kojima H., Nagano T. (2004). J. Am. Chem. Soc..

[cit30] Karolin J., Johansson L. B.-A., Strandberg L., Ny T. (1994). J. Am. Chem. Soc..

[cit31] Paul A., Mandal P. K., Samanta A. (2005). J. Phys. Chem. B.

[cit32] Boydston A. J., Pecinovsky C. S., Chao S. T., Bielawski C. W. (2007). J. Am. Chem. Soc..

[cit33] Fuller J., Carlin R., Simpson L., Furtak T. (1995). Chem. Mater..

[cit34] Fortuna C. G., Bonaccorso C., Qamar F., Anu A., Ledoux I., Musumarra G. (2011). Org. Biomol. Chem..

[cit35] Thakare S. S., Chakraborty G., Krishnakumar P., Ray A. K., Maity D. K., Pal H., Sekar N. (2016). J. Phys. Chem. B.

[cit36] Prakasam M., Anbarasan P. M. (2016). RSC Adv..

[cit37] Avramopoulos A., Zaleśny R., Reis H., Papadopoulos M. G. (2020). J. Phys. Chem. C.

[cit38] Yanai T., Tew D. P., Handy N. C. (2004). Chem. Phys. Lett..

[cit39] Frisch M. , Trucks G., Schlegel H., Scuseria G., Robb M. and Cheeseman J., Gaussian 09, Revision B. 01, Gaussian Inc, Wallingford CT, 2009

[cit40] Karakas A., Elmali A., Unver H. (2007). Spectrochim. Acta, Part A.

[cit41] Lu T. , Software manual. Version, 2014, p. 3

[cit42] Guido C. A., Cortona P., Mennucci B., Adamo C. (2013). J. Chem. Theory Comput..

[cit43] Curioni A., Boero M., Andreoni W. (1998). Chem. Phys. Lett..

[cit44] Hutchison G. R., Ratner M. A., Marks T. J. (2005). J. Am. Chem. Soc..

[cit45] Kutzelnigg W. (1992). Angew. Chem..

[cit46] Hu R., Lager E., Aguilar-Aguilar A., Liu J., Lam J. W., Sung H. H., Williams I. D., Zhong Y., Wong K. S., Pena-Cabrera E. (2009). J. Phys. Chem. C.

[cit47] Chattaraj P. K., Sarkar U., Roy D. R. (2006). Chem. Rev..

[cit48] Gunasekaran S., Balaji R. A., Kumeresan S., Anand G., Srinivasan S. (2008). Can. J. Anal. Sci. Spectrosc..

[cit49] Khalid M., Ali M., Aslam M., Sumrra S. H., Khan M. U., Raza N., Kumar N., Imran M. (2017). Int. J. Pharm. Sci. Res..

[cit50] Zhang H., Yang Y., Xiao H., Liu F., Huo F., Chen L., Chen Z., Bo S., Qiu L., Zhen Z. (2017). J. Mater. Chem. C.

[cit51] Oudar J. d. (1977). J. Chem. Phys..

[cit52] Verbiest T., Houbrechts S., Kauranen M., Clays K., Persoons A. (1997). J. Mater. Chem..

[cit53] Zhang T., Yan L.-K., Cong S., Guan W., Su Z.-M. (2014). Inorg. Chem. Front..

[cit54] Paul S., Misra A. (2011). Inorg. Chem..

[cit55] Yu H.-L., Wang W.-Y., Hong B., Zong Y., Si Y.-L., Hu Z.-Q. (2016). Phys. Chem. Chem. Phys..

[cit56] Patil P. S., Maidur S. R., Shkir M., AlFaify S., Ganesh V., Krishnakanth K. N., Rao S. V. (2018). J. Appl. Crystallogr..

[cit57] Dreuw A., Weisman J. L., Head-Gordon M. (2003). J. Chem. Phys..

[cit58] Le Guennic B., Maury O., Jacquemin D. (2012). Phys. Chem. Chem. Phys..

[cit59] Cai Z.-L., Crossley M. J., Reimers J. R., Kobayashi R., Amos R. D. (2006). J. Phys. Chem. B.

[cit60] Kobayashi R., Amos R. D. (2006). Chem. Phys. Lett..

[cit61] Cheung D. L., Troisi A. (2008). Phys. Chem. Chem. Phys..

[cit62] Sakanoue K., Motoda M., Sugimoto M., Sakaki S. (1999). J. Phys. Chem. A.

[cit63] Kim J. Y., Yasuda T., Yang Y. S., Adachi C. (2013). Adv. Mater..

[cit64] Zhao C., Ge H., Yin S., Wang W. (2014). J. Mol. Model..

[cit65] Geng Y., Li H.-B., Wu S.-X., Su Z.-M. (2012). J. Mater. Chem..

[cit66] Jou J.-H., Kumar S., Agrawal A., Li T.-H., Sahoo S. (2015). J. Mater. Chem. C.

[cit67] Peumans P., Yakimov A., Forrest S. R. (2003). J. Appl. Phycol..

[cit68] Marcus R. A. (1993). Angew. Chem., Int. Ed. Engl..

[cit69] Zou L.-Y., Zhang Z.-L., Ren A.-M., Ran X.-Q., Feng J.-K. (2010). Theor. Chem. Acc..

