# Supplementary material for: The role of π-linkers and electron acceptors in tuning the nonlinear optical properties of BODIPY-based zwitterionic molecules
Source: RSC Adv. 2020 Nov 5;10(66):40300–9. doi: 10.1039/d0ra02193h (PMC9057471; doi:10.1039/d0ra02193h)
Supplement: RA-010-D0RA02193H-s001 [file RA-010-D0RA02193H-s001.pdf]

Electronic Supplementary Material (ESI) for RSC Advances.
This journal is © The Royal Society of Chemistry 2020

Electronic Supplementary Information

The Role of π-Linkers and Electron Acceptors in Tuning the Nonlinear Optical Properties of BODIPY Based Zwitterionic Molecules

Tanushree Sutradhar and Anirban Misra*

Department of Chemistry, University of North Bengal, Darjeeling – 734 013, West Bengal, India

***Phone: +91-9434228745; Email: anirbanmisra@yahoo.com; anirbanmisra@nbu.ac.in**

Item	Description	pages
**Fig. S1**	Optimized structure of compounds 1	S2
**Table S1**	Dihedral angles and bond angles of optimized structure of compound 1	S2
**Fig. S2**	Optimized structure of compound 1a	S2
**Table S2**	Dihedral angles and bond angles of optimized structure of compound 1a	S3
**Fig. S3**	Optimized structure of compound 1b	S3
**Table S3**	Dihedral angles and bond angles of optimized structure of compound 1b	S3
**Fig. S4**	Optimized structure of compound 1c	S4
**Table S4**	Dihedral angles and bond angles of optimized structure of compound 1c	S4
**Fig. S5**	Optimized structure of compounds 1d	S4
**Table S5**	Dihedral angles and bond angles of optimized structure of compound 1d	S5
**Fig. S6**	Optimized structure of compounds 2	S5
**Table S6**	Dihedral angles and bond angles of optimized structure of compound 2	S5
**Fig. S7**	Optimized structure of compound 2a	S6
**Table S7**	Dihedral angles and bond angles of optimized structure of compound 2a	S6
**Fig. S8**	Optimized structure of compound 2b	S6
**Table S8**	Dihedral angles and bond angles of optimized structure of compound 2b	S6-S7
**Fig. S9**	Optimized structure of compound 2c	S7
**Table S9**	Dihedral angles and bond angles of optimized structure of compound 2c	S7
**Fig. S10**	Optimized structure of compound 2d	S7
**Table S10**	Dihedral angles and bond angles of optimized structure of compound 2d	S8
**Table S11**	Computed absorption maxima (λ_{max} in nm), oscillator strengths (*f*), electronic excitation energies (eV) of dyes (1 to 2d) using TD-DFT method at B3LYP/6-311+G(d,p) level.	S8-S9
**Table S12**	Static polarizability, first hyperpolarizability of the studied dyes at the CAM-B3LYP/6-311++G(d,p) level of theory.	S9

**Table S13**	β_x, β_y, and β_z components (10^{-30} esu) of studied molecules (1 to 2d) obtained from DFT calculation (CAM-B3LYP functional), employing the 6-311++G(d,p) basis Set	S9
**Table S14**	Benchmarking of hyperpolarizability.	S10

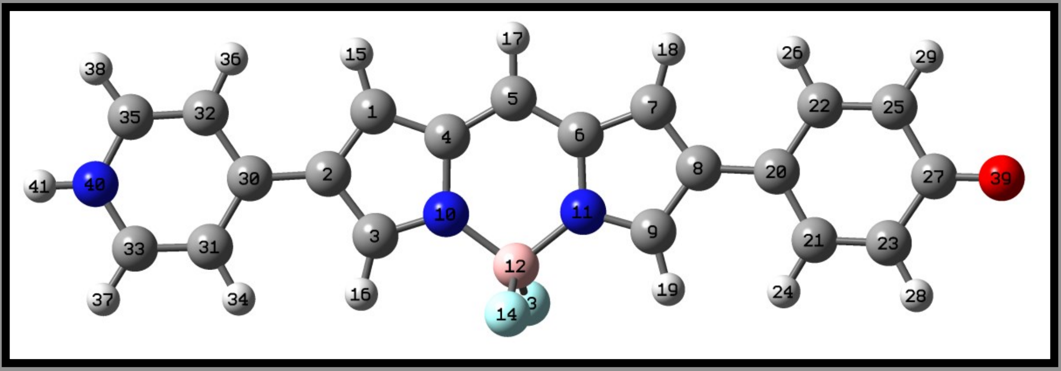

Fig. S1 Optimized structure of compounds 1

Table S1 Dihedral angles and bond angles of optimized structure of compound 1

**Dihedral angles**	
C32-C30-C2-C3	-179.3
C7-C8-C20-C21	178.28
**Bond angles**	
C32-C30-C2	121.2
C31-C30-C2	123.3
C8-C20-C22	120.7
C8-C20-C21	122.2

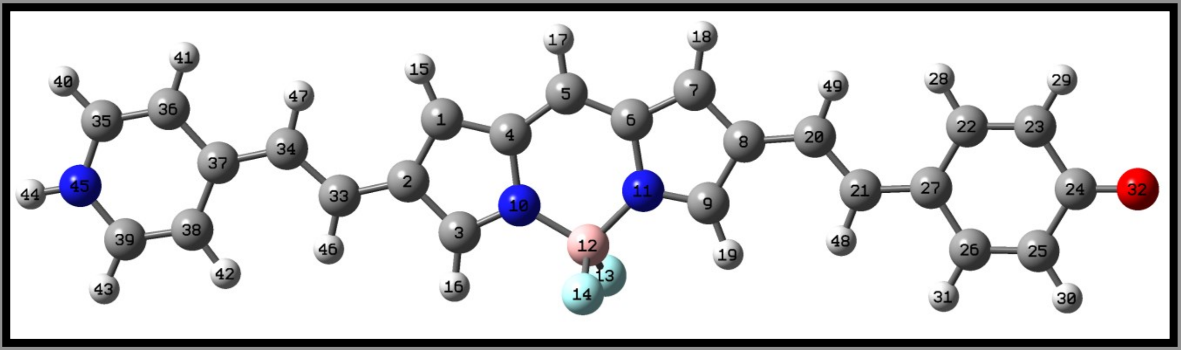

Fig. S2 Optimized structure of compounds 1a

Table S2 Dihedral angles and bond angles of optimized structure of compound 1a

**Dihedral angles**	
C37-C34-C33-C2	-179.9
C20-C21-C27-C26	179.9

Bond angles	
C36-C37-C34	120.4
C38-C37-C34	124.5
C21-C27-C22	123.1
C21-C27-C26	119.2

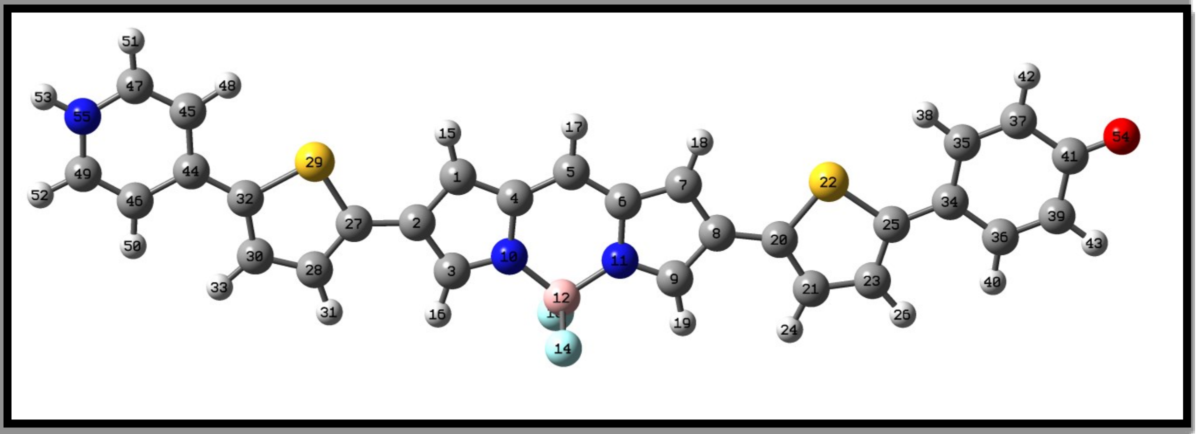

Fig. S3 Optimized structure of compounds 1b

Table S3 Dihedral angles and bond angles of optimized structure of compound 1b

Dihedral angles	
C45-C44-C32-C30	-179.9
C45-C44-C32-S29	0.002
S29-C27-C2-C3	-179.9
C9-C8-C20-S22	179.9
C23-C25-C34-C35	180
S22-C25-C34-C35	-0.003
**Bond angles**	
C44-C32-S29	122.2
C34-C25-S22	122

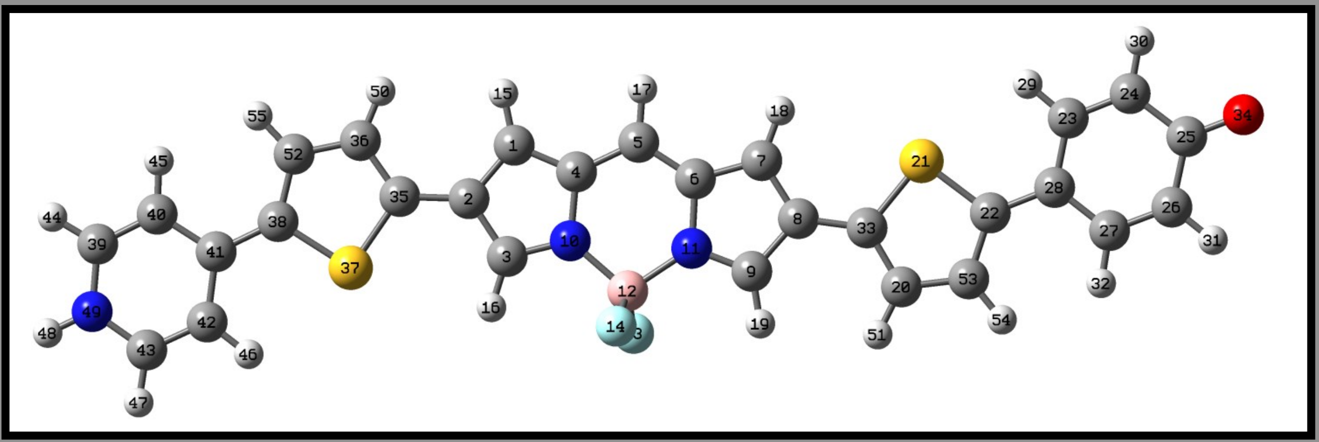

Fig. S4 Optimized structure of compounds 1c

Table S4 Dihedral angles and bond angles of optimized structure of compound 1c

**Dihedral angles**	
C42-C41-C38-C52	179.9
C42-C41-C38-S37	0.001
C36-C35-C2-C3	-179.9
C7-C8-C33-C20	180
C7-C8-C33-S21	-0.001
C53-C22-C28-C23	180
S21-C22-C28-C27	-179.9
**Bond angles**	
C41-C38-S37	122.2
C28-C22-S21	122.1

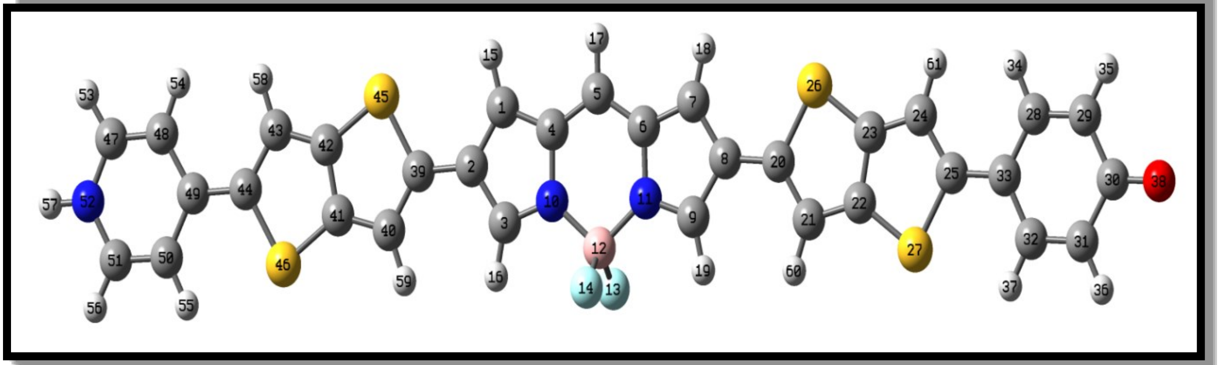

Fig. S5 Optimized structure of compounds 1d

Table S5 Dihedral angles and bond angles of optimized structure of compound 1d

**Dihedral angles**	
C48-C49-C44-S46	179.9
C40-C39-C2-C1	-179.9
S45-C39-C2-C3	-179.9
C7-C8-C20-C21	-180
C9-C8-C20-C26	-179.9
C28-C33-C25-S27	179.9
**Bond angles**	
C49-C44-S45	121.3
S45-C39-C2	120.1
C8-C20-C26	120.3
C33-C25-S27	121.2

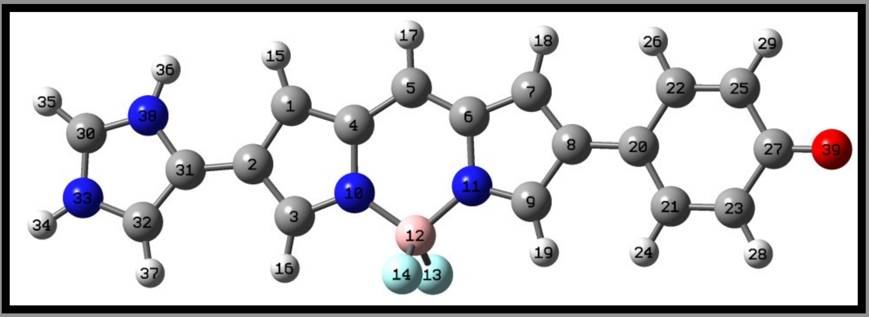

Fig. S6 Optimized structure of compounds 2 in gas phase

Table S6 Dihedral angles and bond angles of optimized structure of compound 2

**Dihedral angles**	
N38-C31-C2-C3	-175.9
C7-C8-C20-C21	178.9
**Bond angles**	
N38-C31-C2	122.3
C8-C20-C21	122.4

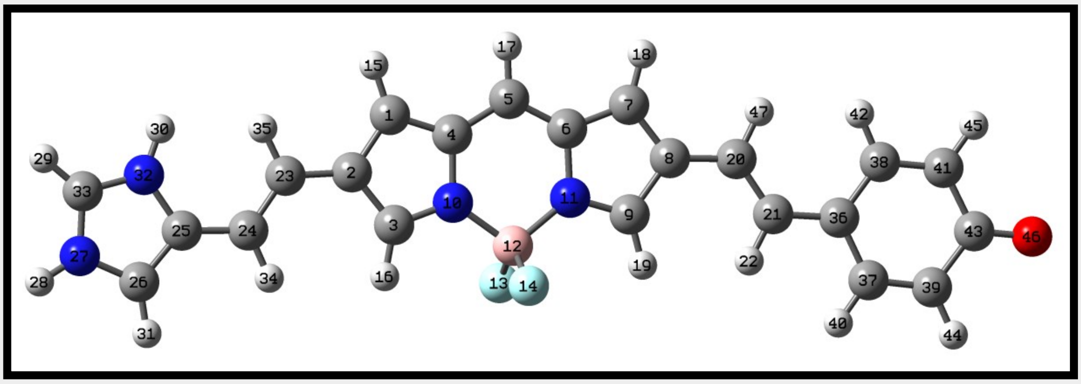

Fig. S7 Optimized structure of compounds 2a in gas phase

Table S7 Dihedral angles and bond angles of optimized structure of compound 2a

**Dihedral angles**	
C25-C24-C23-C2	179.9
C8-C20-C21-C36	-180
**Bond angles**	
N32-C25-C24	125.3
C21-C36-C37	119.3

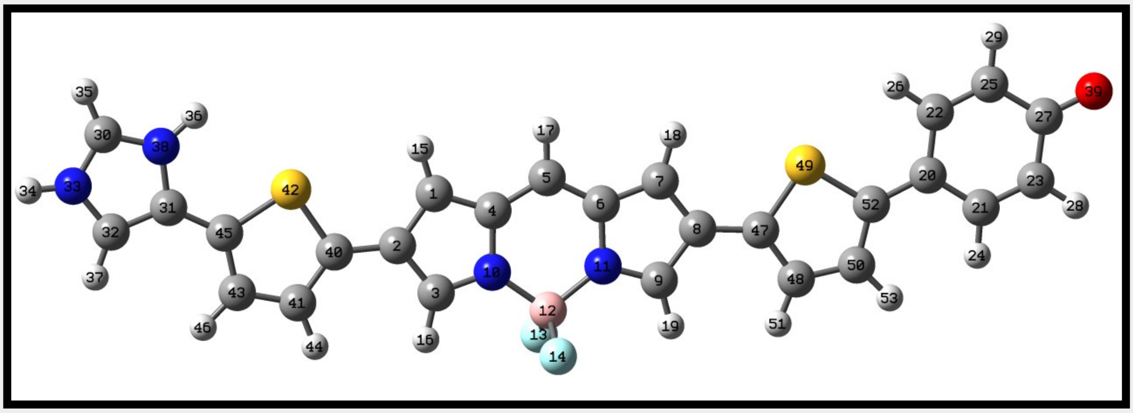

Fig. S8 Optimized structure of compounds 2b in gas phase

Table S8 Dihedral angles and bond angles of optimized structure of compound 2b

**Dihedral angles**	
N38-C31-C45-C43	-179.9
S42-C40-C2-C3	179.9
S49-C47-C8-C9	179.9
C22-C20-C52-C50	180
**Bond angles**	
C31-C45-S42	121.8

S42-C40-C2	120.5
C8-C47-S49	121.4
S49-C52-C20	122.0

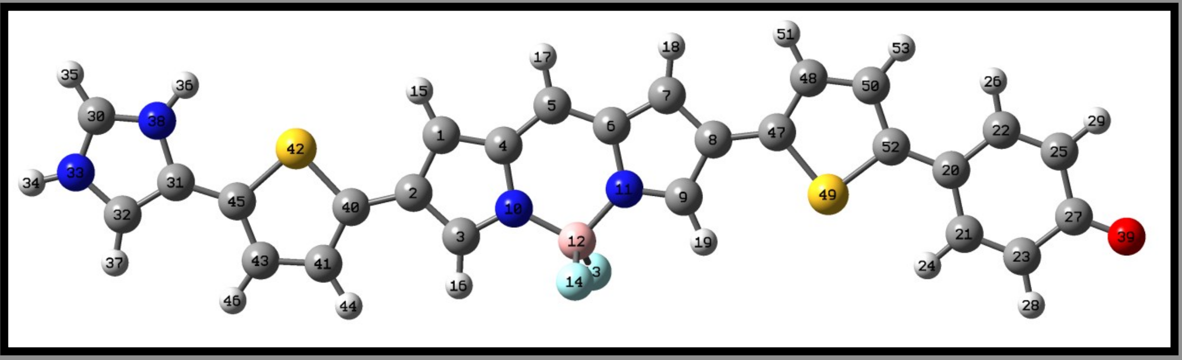

Fig. S9 Optimized structure of compounds 2c in gas phase

Table S9 Dihedral angles and bond angles of optimized structure of compound 2c

**Dihedral angles**	
S38-C31-C45-C43	-180
S42-C40-C2-C3	179.9
C7-C8-C47-S49	179.9
C50-C52-C20-C21	179.9
**Bond angles**	
C31-C45-S42	121.8
S42-C40-C2	120.5
C8-C47-S49	122.8
S49-C52-C20	122.3

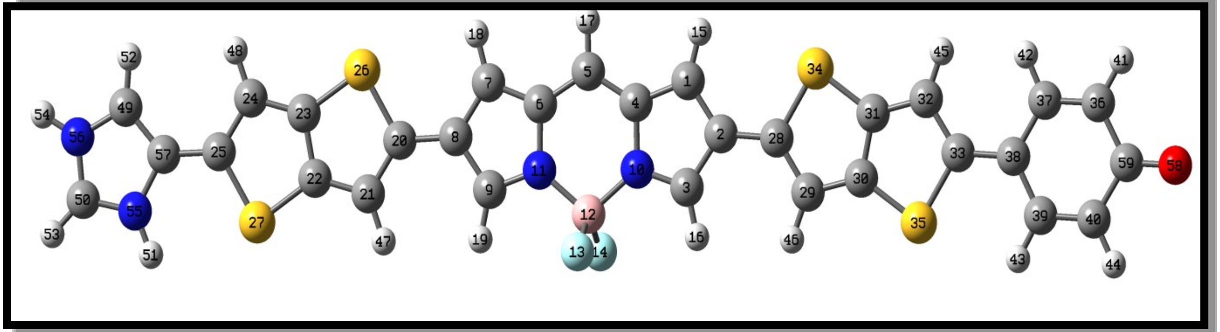

Fig. S10 Optimized structure of compounds 2d in gas phase

Table S10 Dihedral angles and bond angles of optimized structure of compound 2d

Dihedral angles	
N55-C57-C25-C24	-179.9
S26-S20-C8-C9	179.9
S34-C28-C2-C3	-179.9
S35-C33-C38-C37	180
**Bond angles**	
C57-C25-S27	120.9
S26-C20-C8	119.9
C2-C28-S34	120.3
S35-C33-C38	120.1

Table S11 Main electronic transitions, maximum absorption wavelength (λ_{max}) ,oscillator strength(*f*) and transition nature of BODIPY based dyes in gas phase at B3LYP/6-311++G(d,p) level of theory.

Dye	Excited energy (eV)	λ_{max} (nm)	*f*	Assignment	
**1**	1.229	1008.17	0.4718	H → L	0.587
	1.057	1173.07	0.1907	H → L+1	0.519
	1.761	704.18	0.0282	H → L+2	0.697
**1a**	1.1	1126.71	0.7545	H → L	0.397
	1.264	980.96	0.4404	H → L +1	0.491
**1b**	0.953	1301.28	0.9164	H → L	0.374
	1.151	1076.74	0.4941	H → L +1	0.493
**1c**	0.807	1536.02	0.5670	H → L	0.725
	1.155	1073.14	0.7233	H → L + 1	0.634
**1d**	0.735	1684.85	1.209	H → L	0.351
	1.046	1184.92	0.5106	H → L +1	0.509
**2**	0.997	1243.53	0.1438	H → L	0.513
	1.322	938.02	0.3066	H → L+1	0.447
**2a**	0.756	1639.72	0.1830	H → L	0.665
	1.229	1008.30	0.5580	H → L+1	0.568

**2b**	0.729	1701.23	0.1683	H → L	0.452
	1.021	1213.96	0.5388	H → L+1	0.272
**2c**	0.700	1770.09	0.1651	H → L	0.524
	0.969	1279.28	0.7996	H → L+1	0.581
**2d**	0.653	1897.67	0.2899	H → L	0.575
	0.850	1458	0.5713	H → L+1	0.458

Table S12 Static polarizability, first hyperpolarizability of the studied dyes at the CAM-B3LYP/6-311++G(d,p) level of theory.

**Molecules**	**$\Delta\alpha$ 10^{-24} esu**	**β_{total} 10^{-30} esu**
1	107.13	462.05
1a	176.87	2212.40
1b	267.10	4258.66
1c	268.44	5157.16
1d	430.23	7413.08
2	77.19	1023.04
2a	129.89	1967.89
2b	190.94	4572.24
2c	200.71	5492.05
2d	301.62	14,231.26

Table S13 β_x, β_y, and β_z components (10^{-30} esu) of studied molecules (1 to 2d) obtained from DFT calculation (CAM-B3LYP functional), employing the 6-311++G(d,p) basis set.

**Molecul es**	**β_x**	**β_y**	**β_z**
1	2.93	32.82	460.88
1a	1.79	347.97	2184.79
1b	-9.99	281.70	4249.32
1c	93.3	-703.09	5108.17
1d	-48.4	798.11	7639.83
2	-6.72	152.02	1011.66
2a	33.0	-113.76	1963.70
2b	21.2	275.39	4563.89
2c	0.72	203.38	5488.59
2d	-21.1	-729.59	14212.53

Table S14 Benchmarking of hyperpolarizability.

**Molecule**	**Solvent**	**Experiment** β_{total} **(10^{-30} esu)**	**Theoretical** β_{total} **(10^{-30} esu)**	
			B3LYP /6-311++g(d,p)	CAM-B3LYP/ 6-311++G(d,p)
P-Nitroaniline	benzene	34.5	28.82	14.78
Paradimethylamino-*β*-nitrostyrene	Ethanol	220 ± 40	196.55	162.46
